# P50 Addressing the vocational development of young people with long-term health conditions in health care settings: a systematic review and mixed methods synthesis

**DOI:** 10.1093/rap/rkac067.050

**Published:** 2022-09-28

**Authors:** Albert Farre, Laura Lunt, Rebecca Lee, Suzanne Verstappen, Janet McDonagh

**Affiliations:** University of Dundee, Dundee, United Kingdom; Centre for Epidemiology Versus Arthritis, University of Manchester, Manchester, United Kingdom; NIHR Manchester Biomedical Research Centre, Manchester University Hospitals NHS Trust, Manchester, United Kingdom; Centre for Epidemiology Versus Arthritis, University of Manchester, Manchester, United Kingdom; NIHR Manchester Biomedical Research Centre, Manchester University Hospitals NHS Trust, Manchester, United Kingdom; Centre for Epidemiology Versus Arthritis, University of Manchester, Manchester, United Kingdom; Centre for Epidemiology Versus Arthritis, University of Manchester, Manchester, United Kingdom; NIHR Manchester Biomedical Research Centre, Manchester University Hospitals NHS Trust, Manchester, United Kingdom; Department of Paediatric and Adolescent Rheumatology, Royal Manchester Children’s Hospital, Manchester, United Kingdom

## Abstract

**Introduction/Background:**

Long term health conditions (LTHC) such as rheumatic conditions have significant impact on the biopsychosocial development of young people (YP) including vocational development. Educational transitions are prominent during adolescence and young adulthood yet not all transitional care programmes in rheumatology address this area [1]. The aim of this study was to identify and synthesise the benefits and experiences of addressing the vocational development of YP with LTHC in health care settings.

**Description/Method:**

A mixed methods synthesis approach [2] was employed. We systematically searched 10 bibliographic databases. Restrictions were applied on publication date (1996-2020) and publication language (English). Articles reporting quantitative and/or qualitative primary research on addressing vocational needs/issues of YP with LTHC in health care settings were included. YP was defined as 10-24 years [3]. Two reviewers independently screened records using predetermined inclusion/exclusion criteria [4]. Quality appraisal was undertaken following study selection. Qualitative data were synthesised thematically. Quantitative data were synthesised narratively, given that a pooled synthesis was not considered appropriate. A cross-study synthesis integrated findings from both the qualitative and quantitative syntheses.

**Discussion/Results:**

43 articles were included. The quality of qualitative evidence was good; however, the quality of quantitative evidence was poor. The thematic synthesis of stakeholders’ perspectives (n = 23 qualitative studies) resulted in seven recommendations for interventions: provide skills training; provide psychological support; offer to liaise with key stakeholders in educational/workplace settings; provide specialist career advice; provide information, signposting and facilitate access to supporting services; provide/facilitate access to social support; provide flexible care and optimal disease management to support education/employment transitions. The narrative synthesis summarised the results of 17 interventions. The cross-study synthesis mapped interventions against the set of recommendations arising from stakeholders’ perspectives: four interventions met five recommendations; two interventions met four recommendations; five interventions met three recommendations; six interventions met two recommendations. Transitional care interventions were the type of intervention that most comprehensively met the recommendations. The way in which interventions addressed vocational issues was not always clear, with some interventions addressing them directly and others indirectly. No interventions had vocational issues as the core, defining component of the intervention.

**Key learning points/Conclusion:**

Existing stakeholder evidence highlights that vocational development is an important area to address in the care of YP with LTHC such as rheumatic diseases. The resulting set of recommendations provides guidance for future research in this area and transitional care developments in rheumatology. Further work in this area should address these aspects to enable better quality evidence and ensure consistency.

References

[1] Clemente D et al. Pediatr Rheumatol Online J. 2017 Jun 9;15(1):49.

[2] Kavanagh, J et al Synthesizing Qualitative Research: Choosing the Right Approach. Wiley-Blackwell, Chichester, UK, pp. 113–136

[3] World Health Organization, 2001. The second decade: improving adolescent health and development. Geneva.

[4] Farre A et al. PROSPERO 2016 CRD42016051359.

